# Complications Following Percutaneous Mitral Valve Repair

**DOI:** 10.3389/fcvm.2019.00146

**Published:** 2019-10-18

**Authors:** Livia Gheorghe, Alfonso Ielasi, Benno J. W. M. Rensing, Frank D. Eefting, Leo Timmers, Azeem Latib, Martin J. Swaans

**Affiliations:** ^1^Department of Cardiology, St. Antonius Hospital, Nieuwegein, Netherlands; ^2^Department of Clinical and Interventional Cardiology, S. Ambrogio Cardio-Thoracic Center, Milan, Italy; ^3^Department of Cardiology, Montefiore Medical Center, New York, NY, United States

**Keywords:** mitral valve (MV) repair, complications, transcatheter interventions, MitraClip®, Carillon device, Mitralign, Cardioband

## Abstract

Mitral valve disease affects more than 4 million people in the United States and it is the second most prevalent valvulopathy in Europe. The gold standard of treatment in these patients is surgical repair or mitral valve replacement. In the last decade, numerous transcatheter therapies have been developed to overcome the increased number of subjects with symptomatic severe mitral regurgitation and high surgical risk. The Mitraclip (Abbott Vascular, Menlo Park, CA), PASCAL (Edwards Lifesciences, Irvine, CA, USA), the Carillon™ Mitral Contour System™ (Cardiac Dimension Inc., Kirkland, WA, USA), the Mitralign™ (Mitralign, Tewksbury, Massachusetts), and the Cardioband (Edwards Lifesciences, Irvine, CA) are the principal percutaneous devices for mitral valve repair. We present an evidence-based clinical update that provides an overview of these technologies and their potential complications.

## Introduction

The prevalence of mitral regurgitation (MR) is continuously increasing and it became the most prevalent valvulopathy in patients older than 75 years of age in the United States and the second in Europe (1). Surgical repair (when the likelihood of successful repair is high) or replacement is the standard therapy for patients with severe MR (2). Nevertheless, in elderly patients with multiple comorbid conditions, cardiac surgery has a high mortality rate. In the last decade, numerous transcatheter therapies have been developed to overcome the increased number of subjects with symptomatic severe MR and high surgical risk. Percutaneous edge-to-edge procedure- the Mitraclip (MC), with more than 80,000 treated patients is so far, the most well-known percutaneous mitral intervention for MR. Previous trials and studies showed that MC is a safe procedure (3–6). Percutaneous mitral annuloplasty using the Carillon™ and the Cardioband device showed also encouraging results with a low complication rate which can vary from one to another study and it may be related to the operator experience and mitral valve (MV) complexity.

### The Mitraclip Device and New Generation System 3.0

Similar to the previous versions (first-generation and NT), the MC XTR device consists of two main steering components: a 24-F steerable guide catheter (SGC) and a steerable clip delivery system (CDS), with the implant attached at its tip. The rotational knobs on the handles controlling the flexion mechanism of the guide catheter and CDS are similar to the previous versions of the system. The changes made in the clip delivery catheter have the objective of facilitating better stability and minimize unintended translation of the clip during rotation of the CDS. The steerable sleeve has also been adapted to facilitate response to the rotation of the M-knob.

The mechanism and material of the lock line have been modified (braided polyester core surrounded by high–molecular weight polyethylene), enabling the system to be operated in the “unlocked” position.

The substitution of the gripper material from Elgiloy to Nitinol had supposed a higher deeper gripper drop and facilitated the grasping angle.

The new XTR clip is 5-mm longer than the previous generation. The extended arm's length is 22 vs. 17 mm (the older version).

Following femoral vein puncture, adequate access preparation, and transseptal puncture, the SGC is advanced into the right atrium in a straightened position and then inserted 2–3 cm into the left atrium in the neutral position. Once the SGC has been placed, the CDS is inserted and straddled to enable the steering of the device with ease.

Straddling is performed carefully under fluoroscopic and echocardiographic guidance to avoid perforation of the left atrial wall, left atrial appendage, and surrounding structures.

The alignment of the CDS, perpendicular to the mitral coaptation plan is performed and the clip arms (closed up to 60°) are advanced into the left ventricle (LV). The perpendicularity must re-assess before leaflet grasping. Following, both the leaflets “lapping” into the clip arms should be seen ensuring adequate leaflet insertion. Once the final position is achieved, an exhaustive assessment of the result (degree of MR, final mitral gradient) is performed, followed by the clip release.

### Complications During Mitraclip Procedure

Since the first case in 2003 up to now, more than 80,000 MC procedures have been performed. The first trial EVEREST (7, 8) has clearly shown the safety of the device. Moreover, both randomize trials (3, 5, 7, 9) and “real world” registries (4, 6, 10, 11) confirmed that the MC procedure is safe with a high percentage of acute procedural success and minimal complications. The Mitraclip device suffered modifications over time, in order to solve some limitations and potential complications. The presented complications are almost all related to first and second generation of the Mitraclip device and they can be divided into complications related to the catheterization and complications related to the device implantation (Table 1).

**Table 1:** Complications during and after Mitraclip implantation.

**Complications**	**EVEREST phase I (8)**	**EVEREST (7)**	**TCVT (12)**	**GRASP (6)**	**ACCESS-EU (4)**	**TRAMI (10)**	**TVT (11)**	**COAPT (5)**	**MITRA FR (9)**	**Mitra expand (13)**
Type of study	Trial	Trial	Registry	Registry	Registry	Registry	Registry	Trial	Trial	Registry
Year of publication	2005	2009	2014	2013	2013	2015	2017	2018	2018	2019
Used devices	1st gen	1st gen	1st gen	1st gen	1st gen	1st gen	1st gen	1st and 2nd gen	1st and 2nd gen	3rd gen
Number of patients	27	107	628	117	567	828	2952	302	144	107
**Related to the catheterization**										
In-hospital death	0%	0.9%	2.9%	0.9%	3.4%	2.2%	2.7%	Data not available	Data not available	0.9%
Need for resuscitation	0%	Data not available	Data not available	Data not available	1.8%	0.8%	Data not available	Data not available	0%	Data not available
Stroke	0%	0.9%	0.2%	0.9%	0.7%	0.9%	0.4%	0.7%	1.4%	0%
Myocardial infarction	0%	0%	0%	0%	0.2%	0%	0.1%	0%	0%	0%
Pulmonary embolism	0%	0%	0%	0%	0.2%	0%	Data not available	0%	0%	Data not available
Acute renal failure	0%	0%	0%	0%	4.8%	0.7%	Data not available	Data not available	Data not available	1%
Major bleeding requiring transfusion	3%	3.7%	1.1%	Data not available	Data not available	7.4%	3.9%	Data not available	3.5%	1%
Major vascular complications	0%	Data not available	0.7%	Data not available	Data not available	1.4%	1.1%	Data not available	Data not available	Data not available
Pericardial tamponade	0%	2.8%	1.1%	0%	1.1%	1.9%	1%	Data not available	1.4%	0%
Dislocation of existing pacemaker lead	0%	Data not available	Data not available	Data not available	Data not available	0%	Data not available	Data not available	Data not available	Data not available
Endocarditis	0%	0%	0%	Data not available	Data not available	0%	Data not available	Data not available	Data not available	Data not available
**Related to the clip implantation**										
Single-leaflet device attachment	0%	2.8%	Data not available	Data not available	4.8%	2%	1.5%	Data not available	Data not available	4%
Clip embolization	0%	0%	0.7%	Data not available	0	0%	0.1%	Data not available	Data not available	0%
Early partial leaflet detachment^*^	11%	9%	Data not available	Data not available	0.2%	2%	Data not available	Data not available	Data not available	0%
Thrombus formation on clip	0%	Data not available	Data not available	Data not available	Data not available	0.1%	Data not available	Data not available	Data not available	0%
Isolated leaflet damage	0%	Data not available	Data not available	Data not available	Data not available	Data not available	Data not available	Data not available	Data not available	2%
Relevant mitral stenosis	0%	Data not available	Data not available	Data not available	Data not available	0.5%	Data not available	Data not available	Data not available	Data not available
Conversion to open heart surgery	0%	1.8%	0%	0%	0%	0%	0.7%	Data not available	0%	4%
No procedural success^**^	3%	26%	4.6%	0%	9%	3.4%	8.2%	2%	4.2%	7%
Cardiac surgery during the first 30 days	3%	0.9%	0%	0%	Data not available	0.9%	Data not available	Data not available	0%	Data not available

* During procedure or up to 30 days-follow-up.

** *According to the operator criteria*.

### Complications Related to the Catheterization

#### Vascular Complications

**Figure 1:**
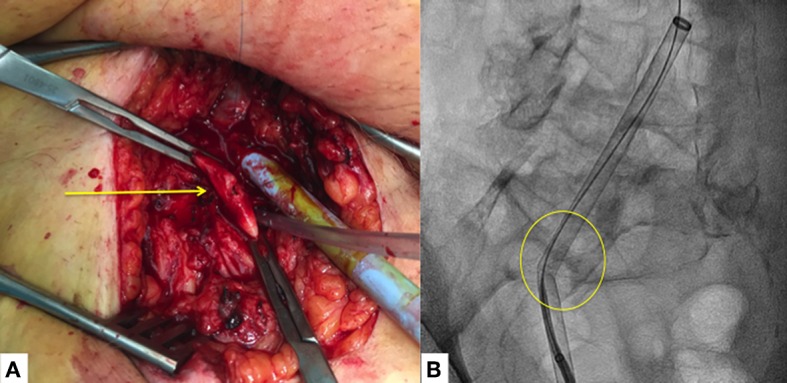
Vascular access complications. **(A)** Major vascular access complication with small laceration of the femoral artery with important fibrotic (adhesions) tissue (yellow arrow). **(B)** Sheath's kinking which does not allow the advance of the transeptal puncture catheter (yellow circle), due to important adhesions between the femoral artery and vein with severe calcified and tortuous iliac artery.

Vascular complications following large-bore venous puncture are infrequent compared to large diameter arterial sheaths (10, 12), nevertheless, optimal access site management in percutaneous MV repair is fundamental. Vascular access complications may occur due to the proximity of the vein to the femoral artery. Inflammatory processes, surgery near the groin may create fibrotic tissue, which could involve both femoral artery and vein. During the 24F sheath's advance, the force applied in the groin may damage the femoral artery (Figure 1). Moreover, fibrotic adhesions between the artery and vein, combined with tortuosity and calcification may impede the sheath's advance and kink (Figure 1). Echo guided puncture, may help to identify the proper access site spot and it can be useful also in cases where the femoral vein is located below the artery. Moreover, due to the elastic venous wall properties, the access site sealing and healing is fast and standard manual compression is an effective and safe method in achieving hemostasis. However, several studies have shown that temporary figure-of-eight suture (Z-suture) is a useful tool in achieving hemostasis by compression of the femoral vein through wrapped and folded subcutaneous soft tissue (14). On the other hand, preclosure suture with the Proglide® (Abbott Vascular Inc., Santa Clara, California) device for larger-sized venous sheaths proved to be safe and allowing an early mobilization (15).

#### Major Bleeding Requiring Transfusion

Although bleeding ranges among the most frequent peri-interventional complications, studies show variable incidences depending on the cohort and definition used, being from 1 to 7.4% (10). It is somewhat intuitive to suspect that bleeding after the MC therapy may arise from the large-caliber femoral venous access, which is required for the 24F guiding sheath. Moreover, a large burden of patients is under anticoagulation therapy and peri-procedural administration of heparin to obtain an activated clotting time (ACT) of more than 250 s increases the risk of access site-related bleeding.

Körber et al. (16) showed in a “real-world registry” that only a third part of the bleedings are related to the access site and the patients with “obscure bleeding” had worse outcomes.

#### Pericardial Tamponade

The risk of pericardial tamponade is low (10) suggesting that transseptal puncture followed by the advancement of the 24F guiding sheath is safe. As in any other procedure in the initial phase of the learning curve, the rate of pericardial tamponade was a little higher (2.8%), reducing to 0% in the recent studies (13). Nowadays, echo guided transseptal puncture aiming to achieve a posterior and superior position is the main key to avoid potential complications. Although the echo guided transseptal puncture, is a straight step during the MC procedure, sometimes it can be challenging in cases of the thick or very floppy septum, post-surgery septum or in cases with chest wall deformities.

#### Ischemic Events: Myocardial Infarction, Pulmonary Embolism, Stroke

Percutaneous MC procedure involves the use of potentially thrombogenic materials through the venous system, transseptal advancement of large-bore catheter devices and beating-heart maneuvering of the clip within complex anatomy of the MV and subvalvular apparatus. However, the rate of the ischemic events as myocardial infarction, pulmonary embolism, and stroke is anecdotic and it is usually multifactorial (Table 1). On the other hand, comparing with other percutaneous structural procedures, the stroke is a rare complication after TMVR; only an incidence of 0.9% of ischemic stroke was documented on 30 days follow-up in the EVEREST RCT trial (3), 2.6% in the EVEREST-HRR (17) and 1.4% in the MTRA-FR trial (9).

Moreover, during the MC device manipulation, there is a small chance of air embolization into the coronary artery (inadequate device preparation), which can produce transitory ischemia that can be treated with high oxygenation and intracoronary nitroglycerine. Thrombus forming within the delivery system can have catastrophic consequences and should be avoided by constantly flushing the catheters as well as by aiming for a high level of anticoagulation during the procedure (the ACT between 250 and 300 s). Some cases with post-interventional thrombus formation in the left atrium (LA) and LV or on the MC device have been reported (18, 19). The prothrombotic state related to thrombus formation into the LA may be produced by the disappearance of severe MR jet agitated blood stasis in LA cavity, endocardial damage during septal puncture, and the duration of the Mitraclip procedure (19).

Although there are not any strict recommendations regarding antiplatelet or anticoagulation regimens post-procedure, patients on anticoagulation treatment continue with it and for the rest of the patients double antiplatelet therapy is encouraged during at least 1 month.

#### Acute Renal Failure

The MC implantation procedure, does not, in itself, require the administration of contrast medium; therefore the acute renal failure is rare. The only study that showed a higher rate of acute renal impairment (4.8% at 30 day follow-up) was ACCESS-EU registry (4), which can be explained by the fact that almost half of the patients presented renal insufficiency at the baseline and it was more prevalent in patients with functional MR and low ejection fraction.

#### Dislocation of Existing Pacemaker Lead

Often, patients with mitral regurgitation, low left ejection fraction, and LBBB require defibrillators or resynchronization therapy implantation, whose cables may interfere during transseptal puncture and SGC advancement. To avoid this potential complication, it is important to double-check with fluoroscopy and echo the relation between the transseptal puncture catheter or GSC and the cables during maneuvering through the right atrium.

#### In-hospital Death and Need for Resuscitation

Even though these are high-risk patients, the procedure itself, has a mortality rate between 0–3.4%. Patients with very low cardiac output, severe right ventricle dysfunction, and severe pulmonary hypertension are more prone to adverse events. Moreover, the available data showed a very low rate of the need for resuscitation.

#### Complications Related to the Mitraclip Device Implantation

Compared with the restrictive inclusion criteria of the EVEREST trial (20, 21), nowadays more patients with challenging anatomy are referred for percutaneous edge-to-edge repair (22). Except for a mitral valve orifice area (MVOA) <4 cm^2^ in COAPT, no specific anatomic exclusion criteria are applied in the most recent randomized trials, MITRA-FR (9) and COAPT (5). The third-generation of the MC device was built to overcome the need to treat even more complex cases with longer, redundant or restricted leaflet and large flail.

It is logical that the greater the complexity of cases, the greater the number of complications, but recent studies did not prove this theory (13). Nevertheless, challenging cases should be done by experienced operators in order to keep procedure safe.

#### Single-Leaflet Device Attachment (SLDA)

It is the most frequent complication with ranges between 0 and 4.8%. SLDA is defined as the loss of the insertion of a single leaflet from the MC device with the ongoing insertion of the opposing leaflet.

It can be acute (during the procedure), subacute (during the first days after the procedure) or late (seen during the follow-up). The majority of the described cases were seen during the procedure and in most cases, it was resolved with second clip implantation.

In the feasibility Everest Trial (7), SLDA occurred in 10 patients (9%), three of them during the procedure, in 1 before hospital discharge, in 5 patients between discharge and 30 days and only 1 partial clip detachment occurred after 30 days. On the other hand, the ACCESS-EU study, which included a large number of patients, showed an SLDA of 4.8%, all most during the first 6 months follow-up. Of these cases, 40% were conservatively managed, another 40% had received another MC device and in 6 cases mitral surgery was needed. Nevertheless, there was no need for urgent surgery or intervention. In the recent, all-comers registry published by Praz et al. (13) the rate of SLDA was also 4%, besides the use of the third-generation MC. The most important step is to perform a meticulous echocardiographic assessment during and after grasping of the leaflets and to ensure proper leaflet insertion into the clip arms.

#### Clip Embolization

**Figure 2:**
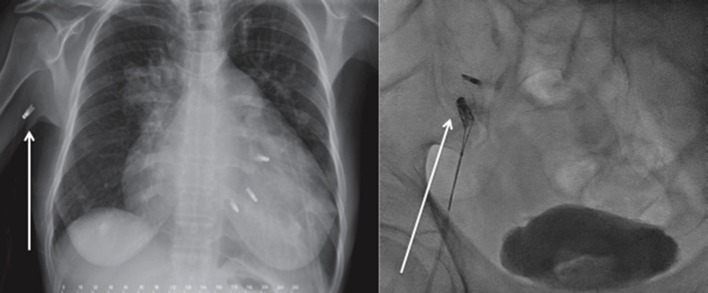
Late Mitraclip embolization. **(Left)** Clip embolization into the axillary artery *Courtesy Dr. Bilge*. **(Right)** Clip embolization into the femoral artery.

MC detachment mostly occurs during the deployment of the clip and is recognized immediately, requiring surgery for its removal. Complex mitral anatomy, several clips implantation with suboptimal echocardiographic window due to the artefacts of the other clips, may be related to clip embolization. Nevertheless, the only two registries that reported clip embolization were the TCVT (12) and the TVT registry [(11); Table 1], whose rate of embolization was <1%. Only a few cases were reported and there are no clear guidelines regarding its management. In late embolization, the clip generally migrates through the arterial system and its removal should be done in case if it induces ischemia [(23–25); Figure 2].

#### Thrombus Formation on the Clip

During the MC procedure, an ACT between 250 and 300 s should be achieved (26). There are no strict recommendations regarding the antiplatelet therapy and in general, the patients do not receive the loading dose. The thrombotic status may determine thrombus formation beside correct anticoagulation, especially in patients with a very low cardiac output and blood stasis.

#### Isolated Leaflet Damage/Tearing

Complex mitral anatomy as severe prolapse, degenerative, or calcified leaflets are more prone to the leaflet damage (27). Sometimes several grasping maneuvers are needed to find a proper clip position, which can damage the ill tissue. Moreover, there are cases where more than one clip is needed to achieve an adequate reduction of MR. Second or third clip implantation is more challenging; because the additional clip is advanced closed in the left ventricle and sometimes the perpendicularity can be lost. In those cases, the clip arms must be everted and withdrawn into the LA. This procedure may harmful and it may produce leaflet tearing or chord rupture. Isolated leaflet damage was described in 2% of patients using the third generation of the MC device (13). In the presence of bigger clip arms, maneuverability is more difficult and there is a higher chance to clip entrapment. Solving isolated leaflet damage is complex, and due to the presence of severe residual mitral regurgitation, in most of cases surgery is required. If the mitral anatomy is favorable (large mitral valve, enough tissue), additional clips can be implanted to stabilize the damaged leaflet. When there is an important gap between the clips, generating a severe mitral regurgitation and another Mitraclip is impossible to implant, an Amplatzer device could be placed to cover the hole (28). Kubo et al. (29), showed in a case series of 9 patients that this technique using an ADO II device is relatively safe and with good results at short time follow-up. Nevertheless, the main complications are device embolization and hemolysis (29).

#### Relevant Mitral Stenosis

In daily practice, it is a common problem for the interventional team to accept a higher transmitral valve gradient for better mitral regurgitation reduction during an MC procedure. TRAMI registry is the only one (10), which presented the rate of relevant mitral stenosis. The rest of the studies just reported the mean transmitral gradient after MC implantation. It is known that patients with relevant mitral stenosis after MV repair had a worse quality of life (30). A mean gradient of more than 5 mmHg is considered not acceptable and it is mainly related to a baseline MVOA <4.0 cm^2^ and with 2 or more clips implantation (31). Before releasing the clip, the echocardiographic assessment is crucial to determine the mitral regurgitation and stenosis. In case of a high mitral gradient with a mitral area <4 cm^2^, the clip should not be implanted. In the other case, in the presence of a high gradient but a mitral area more than 4 cm^2^, the clip should be repositioned. Moreover, continuous left atrial pressure measurement may be useful for decision making during Mitraclip. The mitral regurgitation is correlated with immediate decreases in LA v-wave pressure, LA mean pressure, and left ventricular (LV) end-diastolic pressure (including when LA pressures were indexed to LV pressures to account for changes in afterload) (32). In case of residual MR after implantation of a clip, operators have to decide between clip repositioning or implantation of an additional clip. If the indexed LA mean pressure increases during an additional clip implantation, it should be removed and probably respect the residual MR. If not, an additional clip could be implanted to limit the degree of residual MR.

#### Conversion to Open Heart Surgery

Conversion to open-heart surgery is a rare complication and is it mainly related to the complications mentioned above as clip embolization or MV-injury with severe MR that cannot be treated by clip implantation.

#### No Procedural Success

The rate of no procedural success is between 0–26%. Nevertheless, there is a big variability regarding the definition of no procedural success and it is left to the operator to decide it. The technical success depends on different variables, the mitral anatomy, the operator experience, and the used device. The new device generation is easier to work with and the movements are better transmitted to the clip. After the first feasibility study, the acute procedural success was always more than 90%, besides of more complex cases.

### Pascal Device

The novel Edwards PASCAL transcatheter mitral valve repair (TMVr) system (Edwards Lifesciences, Irvine, CA, USA), similar to Mitraclip device mimics the classical Alfieri stich; nevertheless its design seems to overcome the limitations that have been seen with Mitraclip. The Pascal device improves the reduction of mitral regurgitation through implementation of a central spacer, and allowing for independent leaflet grasping.

It consists of a 10 mm central spacer that acts as filler in the regurgitant orifice of the mitral valve, and is attached to the valve leaflets by two paddles and clasps. The steps of procedure are similar to Mitraclip, with transseptal puncture, aiming a height between 4–5 cm. Nevertheless, the principal advantage of this novel system is the clasps, which can be, operated either simultaneously or independently to facilitate leaflet capture in complex anatomies. The convex curvature of the tip of the paddles aims to reduce tension on the valve leaflets.

### Complications Following PASCAL Transcatheter Mitral Valve Repair System Implantation

Up-to-date only 100 cases with severe mitral regurgitation were performed with the Pascal device. The first-in-man study including 23 patients, showed encouraging results at 30-days follow-up (33). The complications derived from procedure were a minor bleeding and a transient ischemic attack. Cardiovascular morality at 30-days was 9%, and in one case partial leaflet detachment was seen postmortem.

The CLASP Study (NCT03170349) is a multi-center single arm, study to evaluate the safety, performance and clinical outcomes after Pascual device implantation in patients with severe mitral regurgitation. The preliminary results are available in 60 patients. Cardiovascular morality was 1.6%, and without any stroke, myocardial infraction or cardiac tamponade. Severe bleeding was present in 6.5% (n: 4) of patients and only in two of them it was related to the access site complications. Re-intervention was needed in one case (34).

### Carillon System Device

The Carillon^TM^ Mitral Contour System^TM^ (Cardiac Dimension Inc., Kirkland, WA, USA) is a device designed for indirect percutaneous MV annuloplasty through the coronary sinus (CS) of symptomatic patients (NYHA class III-IV despite optimal medical therapy) with dilated cardiomyopathy and moderate-to-severe functional MR. The device received the CE mark in August 2011. The implant features a wire-shaping ribbon (connector), positioned between two interwoven anchors to form a semi-helical shape. The shaping ribbon is designed to be deployed, tensioned, and fastened (percutaneously through the right internal jugular vein- IJV-) inside the CS with the aim to reshape the mitral annulus (MA) favoring leaflet coaptation. Indirect annuloplasty exploits the anatomical position of the CS, which embraces approximately two-thirds of the posterolateral MA from whom it is separated by myocardial tissue. The CS shortening (theoretically) obtained by the tension applied to the device may induce the consequent reduction of the area of the MA. The procedure is performed under general anesthesia and it is fluoroscopic and transoesophageal echocardiography (TOE) guided. An emergency surgical back-up room is needed in case of complications (any emergent conversion was actually reported).

### Complications Following Percutaneous Indirect Mitral Annuloplasty Using the Carillon™ System

The procedure itself is relatively quick (median total procedure time 102 min from first sheath insertion until the last catheter is removed from the body) (35), safe and less invasive compared to other percutaneous MV repair procedures. However, several complications were reported.

### Contrast-Induced Nephropathy (CIN)

The Carillon implantation procedure, does not, in itself, require the administration of a significant amount of contrast medium but several injections of contrast dye are required to assess the anatomical features of the CS, the coronary artery anatomy and its relation to the CS and to guide the implantation of the device within the CS (median contrast volume injection 186 ± 93 ml) (35). Cases of CIN after implantation of the Carillon have been reported (36, 37). Although it is about a severe mitral regurgitation, good hydration or different therapies for kidney protection to avoid CIN may be necessary for patients with renal impairment.

#### Bleedings

The Carillon procedure requires a venous access through the right IJV using a 9F sheath to allow the advancement of a multipurpose catheter (5 or 6F) to selectively cannulate the CS and an arterial access (usually radial with a 6F sheath) to perform a coronary angiogram to assess the relationship between the CS and the coronary three before and during the procedure. As in each percutaneous procedure access, site-related bleedings may occur. To reduce the bleeding risk, an echo-guided IJV puncture should be performed and any vitamin K antagonist oral anticoagulants must be discontinued 3 days before the procedure to achieve an INR between 1.5 and 1.7. In patients under treatment with novel anticoagulants, it is suggested to suspend the treatment 24–48 h before the procedure, depending on the molecule and the renal function of the patient.

**Figure 3:**
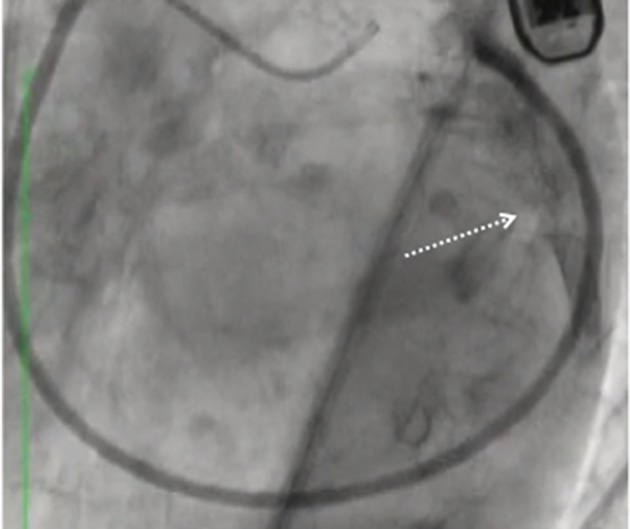
Pericardial effusion (arrow) and cardiac tamponade following the Carillon device delivery system advancement outside the coronary sinus.

**Figure 4:**
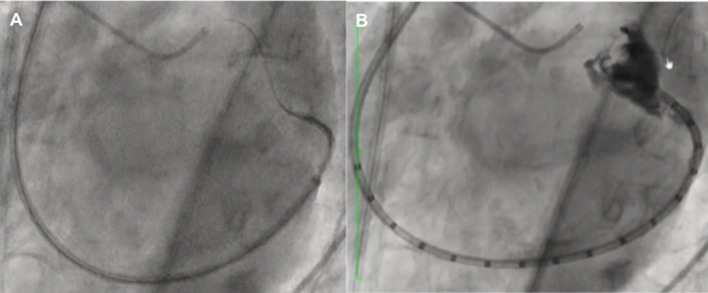
Uncontrolled hydrophilic wire advancement **(A)** leading to coronary sinus perforation **(B)**.

Unlike other MV percutaneous repair procedures (i.e., MC or Cardioband), the Carillon™ procedure does not require transseptal access reducing the risks of interatrial septum puncture-related pericardial effusion or cardiac tamponade (Figure 3). Despite low, the risk of both major bleedings is still present during CS cannulation and guidewire/delivery system advancement within the CS. Perforation or dissection of the CS (3 cases over 48 patients enrolled in the AMADEUS trial (36) may have in the majority of cases a self-limiting course while in the minority may lead to pericardial effusion or cardiac tamponade) (Figure 4). In the latter cases, protamine should be quickly administered to reduce the ACT as much as possible (<200 s) and pericardial drainage should be emergently performed in case of unstable hemodynamic conditions. The possibility to continue the procedure is left, case by case at the operator's discretion according to the patient's hemodynamic stability and general clinical conditions. Complications during the cannulation of the CS are correlated with the learning curve and their rate is similar to that observed in early studies of cardiac resynchronization therapy where CS cannulation is needed (38).

#### Extrinsic Coronary Artery Compression

Given the contiguity of the CS with the coronary arteries, especially the left circumflex artery (LCA), their compression could happen following device deployment and tensioning. A diagonal or ramus branch may have a trajectory between the CS and MA in 16% of patients (39). Special attention should be given to the LCA, which runs between the CS and the MA, in a high percentage, ranging between 64 and 80% of cases (40–42). The LCA may suffer frequently extrinsic compression due to this close relationship with the CS. Moreover, the LCA branches may be also potentially involved (43). If the Carillon™ device is in close relation with a coronary artery segment with a previously implanted stent, it's deployment should de aborted due to the potential compromise of the stent integrity.

A simultaneous CS venogram (left anterior oblique 30° projection) and coronary angiogram at the start of the procedure and coronary angiogram just before the release of the device are mandatories to assess eventual coronary artery extrinsic compression. In case of significant coronary narrowing due to indirect compression, the tension of the implanted device must be reduced and/or the Carillon™ system could be retrieved through a specific capture system to be repositioned (44). In the case of persistent compression (despite tension reduction or device repositioning) associated with EKG modifications, the implantation procedure must be aborted (17% of cases reported in the TITAN II trial) (35).

#### Partial Device Dislodgment/Fracture

The first generation of the device suffered modifications (the shape of the anchors are twisted at the apex providing more rigidity) due to some reported cases of slippage of the distal anchor, which prevented the final release of the implant. One case of device fracture (not fatigue-related) was seen in the TITAN II trial in a patient in whom the device could not be recaptured, leaving a recaptured/redeployed, damaged proximal anchor in the middle of the great cardiac vein at the site of dynamic venous compression. The fracture was not associated with a clinical event (35).

#### Reduced Strength of the Metal

Another potential complication during Carillon™ implantation can be the reduced strength of the metal (nickel and tantalium composing the shaping ribbon between the anchors of the device), which can fail the device in terms of mitral regurgitation degree reduction. With the second generation of the device, used in the TITAN study (37), the number of cases with device failure was significantly reduced and the outcome was improved.

No complete device embolization/dislodgment, procedure-related infections, conduction abnormalities, or iatrogenic mitral stenosis were reported until now following Carillon™ implantation.

### Mitralign™ System Device and Potential Complications

Mitralign (Mitralign, Tewksbury, Massachusetts) is a direct annuloplasty system that uses radiofrequency energy to penetrate sutures for two bident pledgets into the MA tissue posterior and anterior to the commissure (both, atrial and ventricular sides). By cinching the sutures, the MA becomes reduced. The procedure is performed under general anesthesia, guided by 2- and 3-dimensional TOE and fluoroscopy, it requires arterial femoral access, and 14F deflectable guiding catheter manipulation within the LV. The procedural steps were extensively described elsewhere (45) while the procedure aims to reduce the degree of functional MR in the symptomatic patient by the reduction of the MA dimension. Data from the first-in-man trial on 71 high-risk patients demonstrated positive results in terms of LV reverse remodeling, and clinical improvement during 6 months after treatment (46). The main procedural complications reported were cardiac tamponade and access site bleedings (46).

Pericardial tamponade occurred in 4 patients (8.0%) and was managed uneventfully with pericardiocentesis in all the cases with no need for emergency cardiac surgery. Three of the 4 tamponades were related to catheter manipulation within the LV. One of the tamponades led to the exclusion of LV end-diastolic diameter *r* < 5.0 cm while 2 of them were a function of the early learning curve and first-generation devices. Thus, the exclusion of LV end-diastolic diameter <5.0 cm and second-generation catheter systems have mitigated potential risks of tamponade.

Concerning arterial access, there were 6 (8.4%) bleeding complications reported. Three of the complications required transfusion and 3 did not. All were managed conservatively without the need for surgery or interventional repair/stent placement (46).

### Cardioband™ Device

The Cardioband (Edwards Lifesciences, Irvine, CA) is a device designed to perform direct percutaneous annuloplasty (supra-annular fixation like in surgery) of symptomatic patients (NYHA II-IV) with dilated cardiomyopathy and moderate-severe functional MR (due to MA enlargement) by means of a half-ring implanted in the posterior MA, with beating heart, and under fluoroscopic and TOE guidance. Aim of this procedure is to reduce MR by annular reduction. The device and the procedure have been previously described elsewhere (47).

Briefly, the Cardioband implant is a polyester sleeve with radiopaque markers spaced 8 mm apart containing a pre-mounted contraction wire connected to an adjusting spool. The device is fixed *in situ* thanks to a series of helical stainless steel implantable anchors and is equipped with a system that allows adjustment of the degree of annular reduction to achieve a good result in terms of residual MR, without creating stenosis.

The procedure is performed under general anesthesia through venous femoral access and a 25F transseptal steerable sheath (47). Pre-procedural CT scan is mandatory to exclude patients with anatomical contraindications (“superficial” LCA, MA calcification, small left atrial chamber). Furthermore, a simulation of the entire procedure is carried out at the core lab using cardiac CT to plan the number of anchors that need to be released to cover from the anterior area of the lateral commissure toward the posterior area of the medial commissure of the posterior MA.

The optimal position of the transseptal puncture is also determined off-line by CT analyses for each patient and the puncture is echo-guided during the procedure. Encouraging clinical results on 60 patients at 1-year follow-up was recently published (48), even though several complications were described (46, 47).

### Complications Following Direct Percutaneous Mitral Annuloplasty Using the Cardioband™ Device

#### Peri-Procedural Stroke

Cardioband implantation is a relatively long-procedure (total procedural time and device implantation time 201 ± 58 min and 175 ± 50 min, respectively) (48) and different materials (steerable sheath, anchors, band) are manipulated within the left atrium. On this basis, heparin administration is fundamental to maintain an ACT between 250 and 300 s to ensure adequate patient anticoagulation avoiding thrombo-embolic complications. Following implantation, no oral anticoagulation is needed and the dual antiplatelet therapy regimen is indicated according to prior cardiovascular events/procedures. Despite a careful intra/peri-procedural anticoagulation management, one immediate post-procedural, non-fatal ischemic stroke was reported in 1 over 60 patients while one fatal hemorrhagic stroke, few days after device implantation was described in a patient being treated by triple anticoagulant therapy (aspirin, ticagrelor, heparin, and vitamin K antagonist) because of a recently implanted coronary stent and atrial fibrillation (48).

#### Left Circumflex Artery Injury

**Figure 5:**
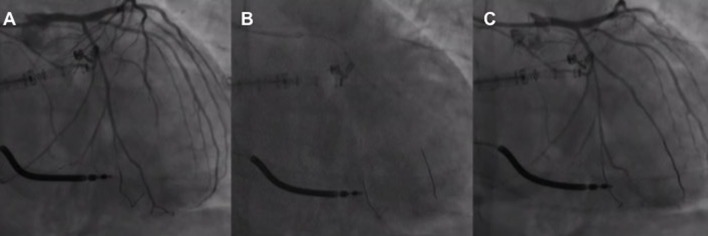
First obtuse marginal narrowing associated with the slow flow **(A)** following implantation and release of the first Cardioband anchor managed with non-compliant balloon inflation **(B)** with narrowing resolution and flow restoration **(C)**.

LCA injury (obstruction or perforation) secondary to anchor placement was reported in 2 over 60 cases (48). This is a well-known complication of mitral valve surgery/intervention (Figure 5). Since then, the screening process for the Cardioband procedure has improved based on CT evaluation assessing the distance between the myocardial surface at the theoretical anchor releasing zone and the LCA. Furthermore, a procedural coronary angiography is recommended before inserting and releasing anchors, especially for the first anchors due to the proximity of the LCA to the MA near the lateral commissure. In the largest report of Cardioband treated patients, LCA injuries have been associated in one case with myocardial infarction while in the other with cardiac arrest due to ventricular rhythm disturbance. Both the events were successfully solved and the patients survived the events (48).

Transient LCA occlusion due to cinching-related coronary kinking despite avoiding injury by the anchor was also reported. Cinching reduction (from 4.5 to 3.5 cm) and stent implantation at the proximal LCA have been adopted as solutions to avoid LCA kinking resolving the acute ischemic myocardial damage (49).

#### Anchor Disengagement

This complication may lead to partial device detachment which might impact device efficacy with significant MR recurrence (47, 48) but any device migration, embolization nor intravascular hemolysis was reported associated with this phenomenon. Since anchors are delivered through the sleeve, if disengaged, they remain within the band and there is theoretically no risk of anchor migration or embolization. No late (more than 30 days) disengagements were reported even if one case of subacute (after 3 days) dehiscence across P2 with 5 anchors disengagement leading to MA laceration, severe MR recurrence and cardiogenic shock requiring Cardioband surgical explantation and left ventricular assist device positioning was recently described (50). Improper or insufficient anchor insertion and a prior shift in the manufacturing process were advocated as potential causes of all incidents of anchor disengagement.

Important improvements were performed to overcome this potential complication. Anchor length was increased from 4 to 6 mm, giving more stability and better anchoring within the myocardium. During cinching the lateral commissure area gives important support, and additional anchors were used to reinforcing this area. The improvement of the imaging techniques, using multiples views made the procedure safer, paying special attention during the pull test in the P2 area (second area at risk for disengagement). The device design was improved to avoid contraction failure, which also occurred early in the series.

As with other devices, the learning curve is important. Indeed, 9 of the 10 anchor disengagement (5 resulting in device inefficacy) occurred in the first 28 patients enrolled in the CE mark trial (47).

**Figure 6:**
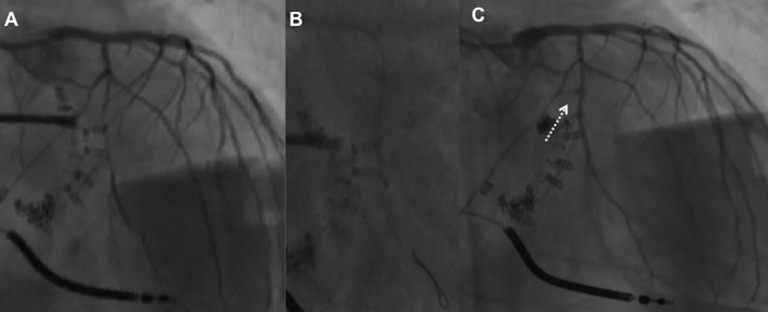
Flow reduction on distal left circumflex **(A)** after the third anchor placement managed by balloon inflation **(B)** which caused anchor detachment with residual coronary-left atrial fistula (**C**, arrow) managed conservatively (covered stent did not advance through the left main toward the left circumflex) with the resolution at 1 month.

Training of both interventional cardiologists and echocardiographers is crucial to reduce this complication and to increase the device success rate [(48); Figure 6]. However, due to the risk of delayed (subacute) dehiscence close echocardiographic controls are of paramount importance at follow-up.

#### Conduction Disorders

Despite the proximity of the deployed ring to the atrioventricular (AV) conduction system, only one case of complete AV block has been reported until now (51). In particular, a late-onset (26 h after the procedure) Mobitz 2 AV block then evolved to complete AV block (in the following day), requiring definitive biventricular pacemaker (PM) was described in an 80-year-old patient with prohibitive surgical risk, treated with Cardioband implantation (17 anchors) for functional MR.

The sub-acute AV block, without any electric disturbance during implantation or cinching is difficult to be explained while the late presentation might be related to the pressure exerted during heart contraction causing permanent damage around the screws where the conduction system is located.

Manipulation of the CS area during different transcatheter interventions may affect the AV conduction system, which is in the vicinity of the CS. Although just one case of late AV block was described, this event should not be generalized and considered as a frequent complication after Cardioband. Moreover, prolonged EKG monitoring after a similar procedure with CS area manipulation should be considered (51).

#### Acute Impairment of Left Ventricular Systolic Function

Similarly to other percutaneous mitral repair procedures performed in patients with functional MR (52), acute impairment of left ventricular (LV) systolic function (afterload mismatch) may occur even after Cardioband procedures (48). Although this phenomenon is usually transient (without long-term prognostic implications) and less frequent compared to surgical MV repair, inotropic drugs may be required to support the circulation. However, it is well-known that β-adrenergic agonists (i.e., dobutamine, adrenaline, and dopamine) may favor on the other hand myocardial ischemia, arrhythmias and increase mid-term mortality in patients with severe LV dysfunction (53).

In this setting, the administration of levosimendan 0.01 μg/kg/min before, during and after the procedure might help to reduce the risk of acute hemodynamic worsening following percutaneous functional MR correction (54).

#### Implant Contraction Failure

This complication may occur in the last phase of the procedure.

After the deployment of the last anchor and the removal of the implant delivery system (IDS), the size adjustment tool (SAT) is then inserted through the trans-septal steerable sheath (TSS), over the implant guidewire, until its distal end reaches the adjustment spool of the implant. After the SAT connection, the implant is contracted by clockwise rotation of the adjustment roller (47). Adequate reduction of MR severity is assessed by TOE under beating heart conditions. When the appropriate implant size has been reached, the SAT is detached from the adjustment spool leaving the implant with the desired degree of contraction.

**Figure 7:**
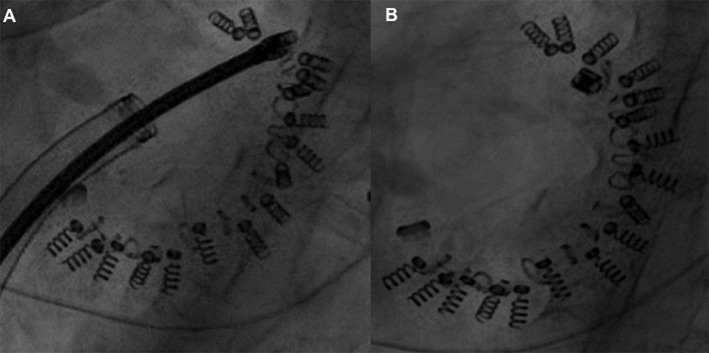
**(A,B)** Guidewire rupture and implant contraction failure with loose of the cinching initially acquired.

In the early experience, two cases of residual significant MR were described and related to the impossibility to contact the Cardioband after the implantation because of technical device failure (Figure 7). This device-related failure was solved with an iteration of the device after the first initial experience (10 patients treated) (47).

#### Contrast-Induced Nephropathy (CIN)

The Cardioband implantation procedure itself does not require the administration of contrast medium as anchors positioning is performed under 3D TOE guidance. However, several injections of contrast dye might be needed to assess the coronary artery anatomy and the relationship between the LCA and the first anchors implanted. Two cases of CIN (over 60 patients reported) after Cardioband implantation have been reported (48).

Therefore, considering that 75% of the patients treated had renal insufficiency before the procedure, good hydration or the use of other means to protect against CIN may be necessary case-by-case according to the clinical features of the patient.

#### Other Serious Adverse Events

Other events reported during (or after) Cardioband implantation were: 2 pericardial effusion (possibly related to procedure), 1 left femoral pseudoaneurysm (related to procedure), 1 bleeding complication (related to procedure), 1 upper limb hemiparesis, 1 gastrointestinal bleeding, 1 late mitral valve endocarditis (47, 48).

No complications (i.e., cardiac tamponade, iatrogenic atrial septal defects) directly related to the trans-septal puncture/access were reported.

## Conclusions

Nowadays, patients with severe MR and high risk for surgery have the percutaneous option for mitral valve repair with a low risk of potential complications.

With all-new technologies, the team must be aware of the procedure, with the complications that may occur and how they can solve it.

## Author Contributions

All authors listed have made a substantial, direct and intellectual contribution to the work, and approved it for publication.

### Conflict of Interest

The authors declare that the research was conducted in the absence of any commercial or financial relationships that could be construed as a potential conflict of interest.
